# The REal Life EVidence AssessmeNt Tool (RELEVANT): development of a novel quality assurance asset to rate observational comparative effectiveness research studies

**DOI:** 10.1186/s13601-019-0256-9

**Published:** 2019-03-27

**Authors:** Jonathan D. Campbell, Robert Perry, Nikolaos G. Papadopoulos, Jerry Krishnan, Guy Brusselle, Alison Chisholm, Leif Bjermer, Michael Thomas, Eric van Ganse, Maarten van den Berge, Jennifer Quint, David Price, Nicolas Roche

**Affiliations:** 1 Center for Pharmaceutical Outcomes Research, University of Colorado, Anschutz Medical Campus, Aurora, CO USA; 2 Allergy Department, 2nd Pediatric Clinic, University of Athens, Athens, Greece; 3 Division of Infection, Immunity and Respiratory Medicine, University of Manchester, Manchester, UK; 4 Population Health Sciences Program, University of Illinois Hospital and Health Sciences System, Chicago, IL USA; 5 Department of Medicine, University of Illinois at Chicago, Chicago, IL USA; 6 Department of Respiratory Medicine, Ghent University Hospital, Ghent, Belgium; 7 Departments of Epidemiology and Respiratory Medicine, Erasmus Medical Center, Rotterdam, The Netherlands; 8 Respiratory Effectiveness Group, Cambridge, UK; 9 Department of Respiratory Medicine and Allergology, Institute of Clinical Science, Lund University, Lund, Sweden; 10 Primary Care and Population Sciences, University of Southampton, Southampton, UK; 11 Pharmacoepidemiology Unit, UMR CNRS 5558, University of Lyon, Lyon, France; 12 Department of Pulmonary Diseases, University Medical Center Groningen, University of Groningen, Groningen, The Netherlands; 13 Respiratory Epidemiology, Occupational Medicine and Public Health, National Heart and Lung Institute, Imperial College London, London, UK; 14 Academic Primary Care, University of Aberdeen, Aberdeen, UK; 15 Observational and Pragmatic Research Institute, Singapore, Singapore; 16 Hôpital Cochin (APHP), University Paris Descartes (EA2511), Paris, France

**Keywords:** Asthma, Comparative effectiveness research (CER), Quality, Observational studies, Assessment tool

## Abstract

**Background:**

Evidence from observational comparative effectiveness research (CER) is ranked below that from randomized controlled trials in traditional evidence hierarchies. However, asthma observational CER studies represent an important complementary evidence source answering different research questions and are particularly valuable in guiding clinical decision making in real-life patient and practice settings. Tools are required to assist in quality appraisal of observational CER to enable identification of and confidence in high-quality CER evidence to inform guideline development.

**Methods:**

The REal Life EVidence AssessmeNt Tool (RELEVANT) was developed through a step-wise approach. We conducted an iterative refinement of the tool based on Task Force member expertise and feedback from pilot testing the tool until reaching adequate inter-rater agreement percentages. Two distinct pilots were conducted—the first involving six members of the Respiratory Effectiveness Group (REG) and European Academy of Allergy and Clinical Immunology (EAACI) joint Task Force for quality appraisal of observational asthma CER; the second involving 22 members of REG and EAACI membership. The final tool consists of 21 quality sub-items distributed across seven methodology domains: Background, Design, Measures, Analysis, Results, Discussion/Interpretation, and Conflict of Interest. Eleven of these sub-items are considered critical and named “primary sub-items”.

**Results:**

Following the second pilot, RELEVANT showed inter-rater agreement ≥ 70% for 94% of all primary and 93% for all secondary sub-items tested across three rater groups. For observational CER to be classified as sufficiently high quality for future guideline consideration, all RELEVANT primary sub-items must be fulfilled. The ten secondary sub-items further qualify the relative strengths and weaknesses of the published CER evidence. RELEVANT could also be applicable to general quality appraisal of observational CER across other medical specialties.

**Conclusions:**

RELEVANT is the first quality checklist to assist in the appraisal of published observational CER developed through iterative feedback derived from pilot implementation and inter-rater agreement evaluation. Developed for a REG-EAACI Task Force quality appraisal of recent asthma CER, RELEVANT also has wider utility to support appraisal of CER literature in general (including pre-publication). It may also assist in manuscript development and in educating relevant stakeholders about key quality markers in observational CER.

**Electronic supplementary material:**

The online version of this article (10.1186/s13601-019-0256-9) contains supplementary material, which is available to authorized users.

## Background

Asthma affects an estimated 334 million people worldwide, 14% of the world’s children and 8.6% of young adults [1]. With such high global prevalence, the affected patient population is inevitably broad and heterogeneous. Such heterogeneity is observed through level of asthma control, severity, various marker phenotypes, and asthma treatment. However, there remains unknown precipitants of future impairment and risk. Healthcare professionals need informed clinical guidelines to help support decision making and to tailor management approaches to the complex needs of the diverse range of patients seen in clinical practice.

Current asthma guidelines are developed by experts with the goal of signposting evidence-based approaches that will optimize patient outcomes. Their recommendations are based on a synthesis of the available literature and (reflecting traditional evidence hierarchies) rely heavily on classical randomized controlled trials (RCTs). RCTs have relatively high internal validity and the ability to clearly establish a causal relationship between an exposure and an outcome [2]. This high internal validity is achieved through selective patient inclusion and highly-controlled ecologies of care. However, it also limits RCTs’ external validity and their relevance to real-world care and many routine clinical scenarios [3, 4]. In everyday practice, healthcare professionals often encounter complex clinical, lifestyle, psychosocial, demographic and attitudinal factors; aspects of the real world that are excluded as much as possible within RCTs to maximize their internal validity. The clinical implications of these factors are better addressed through real-life research methodologies—observational studies, pragmatic trials, observational comparative effectiveness research (CER)—which, by design, reflect true ecologies of care and include factors such as gradations of disease severity, diverse patient demographics, comorbid conditions, treatment adherence and patients’ lifestyles. Thus, the questions addressed through RCT and CER research are related yet distinct, each having importance toward improving patients’ health. The integration of robust evidence from both RCTs and real-life CER can provide a more complete picture of outcomes in complex clinical scenarios. A combination of evidence sources better represents the totality of an intervention’s effectiveness and guidelines based on such evidence combinations are more applicable to real-world clinical practice and its many and varied associated challenges.

Calls for better integration of a range of evidence sources to inform more holistic respiratory guidelines [5–7] will only be realized if confidence in non-RCT evidence is increased. Inherent difficulties in assessing whether real-life evidence is of sufficient quality to be considered within the context of clinical guidelines has been a barrier to acceptance. Development of a systematic and implementable approach to real-life CER quality appraisal is an important step towards implementing this necessary paradigm shift. Responding to this need, the Respiratory Effectiveness Group (REG) and European Academy of Allergy and Clinical Immunology (EAACI) formed a joint task force to conduct a quality appraisal of the published observational asthma CER literature and, in order to do so, worked systematically to develop a standardized tool for CER quality appraisal (Additional file 1: Fig. 1).

This paper outlines the methodology and process that lead to the development of the REal Life EVidence AssessmeNt Tool (RELEVANT). The tool was created to assist the Task Force in identifying CER studies of sufficiently high quality to warrant consideration by asthma guideline bodies [8].

## Methods

**Fig. 1:**
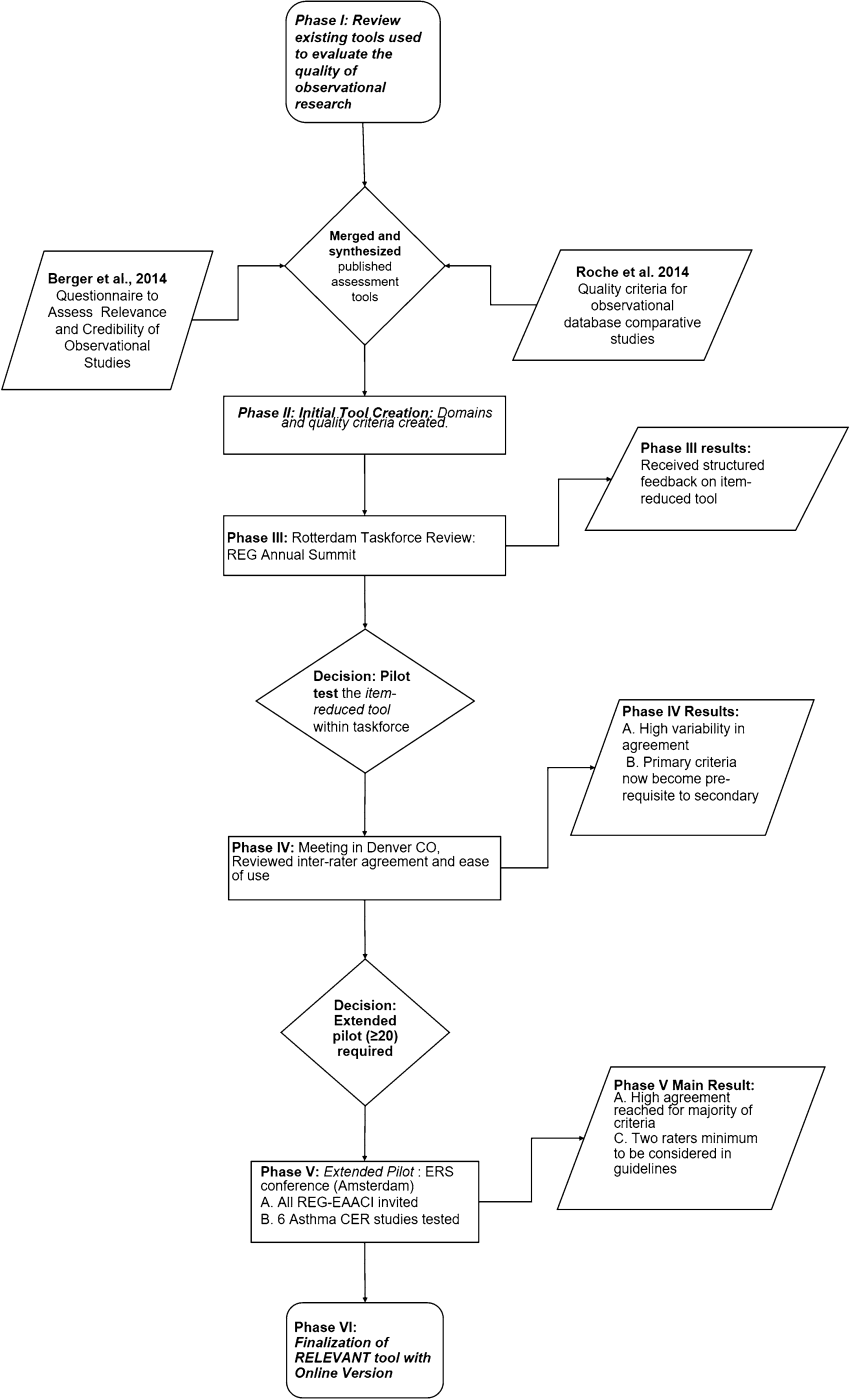
Six key development phases of RELEVANT

RELEVANT was developed across six key phases between June 2014 and September 2015, including a literature review and synthesis of the existing literature followed by iterative processes for creating a novel tool (Fig. 1).

### Phase I: Review of the quality assessment literature

Seeking to integrate the Task Force work within existing quality appraisal activities, a number of publications on quality parameters for non-RCT CER were first identified and reviewed [9–14]. The principles of these publications were chiefly incorporated within two key papers published in 2014—Berger et al. [14] and Roche et al. [13]—which jointly formalize the CER nomenclature and offer recommendations for systematic quality appraisal of observational database studies. Roche et al. [13]—*Quality criteria for observational database comparative studies*—was a literature-based proposal published by members of REG offering suggestions for the quality appraisal of observational database studies with a particular focus on respiratory medicine. Berger et al’s [14] *Good Practice Task Force Report* was developed to evaluate the quality of pharmaceutical outcomes research evidence and was published on behalf of the International Society For Pharmacoeconomics and Outcomes Research (ISPOR), Academy of Managed Care Pharmacy (AMCP) and the National Pharmaceutical Council (NPC). It involved a review of the current literature on reporting standards for outcomes research published prior to 2014 and proposed a number of study design criteria to assist in the assessment of CER within the context of informing healthcare decision making. These two papers were used to identify the quality domains and critical sub-items of these domains that the REG–EACCI Task Force members should incorporate in RELEVANT.

### Phase II: Initial tool creation

The RELEVANT quality domains and sub-items were identified by mapping the components both unique and common to the Berger [14] and Roche [13] publications. The mapping enabled a broad net to be cast such that all domains and quality sub-items were captured while also enabling identification of areas of agreement (core concepts) and redundancies (for elimination).

A structured taxonomy was developed such that design categories common to both papers were defined as quality “Domains” and within these were labeled “sub-items” (markers of methodological quality).

### Phase III: Rotterdam task force review and feedback

The checklist produced (Additional file 2: Table 1) was reviewed and discussed by Task Force members at a face-to-face meeting as part of the REG Summit (Rotterdam, the Netherlands) in January 2015. The central recommendation was to reduce the number of items in the tool to minimize potential inter-rater variability. It was also agreed that all Task Force members would, remotely and independently, review the individual quality sub-items within the checklist and advise whether sub-items should be kept, removed or merged and the rationale for their recommendation. This feedback was used to item-reduce the checklist for initial pilot testing by Task Force members (Table 1).

**Table 1:** Phase III item-reduced checklist assessed using the within-Task Force pilot

Primary or secondary	Items	Score 1 = ”yes”; 0 = ”no”	Reviewer comments
*Background/relevance: 3 items (1 primary, 2 secondary)*			
Primary	1. Clear underlying hypotheses and specific research questions		
Secondary	2. Relevant population and setting		
Secondary	3. Relevant interventions and outcomes are included		
*Design 4 items: (2 primary, 2 secondary)*			
Primary	1. Evidence of a priori protocol, review of analyses, statistical analysis plan, and interpretation of results		
Primary	2. Comparison groups justified		
Secondary	3. Registration in a public repository with commitment to publish results		
Secondary	4. Data sources that are sufficient to support the study^a^		
*Measures: 4 items (2 primary, 2 secondary)*			
Primary	1. Was exposure clearly defined, measured and (relevance) justified^b^		
Primary	2. Primary outcomes defined, measured and (relevance) justified^b^		
Secondary	3. Length of observation: Sufficient follow up duration to reliably assess outcomes of interest and long-term treatment effects		
Secondary	4. Sample size: calculated based on clear a priori hypotheses regarding the occurrence of outcomes of interest and target effect of studied treatment versus comparator		
*Analyses 3 items (1 primary, 2 secondary)*			
Primary	1. Thorough assessment of and mitigation strategy for potential confounders		
Secondary	2. Study groups are compared at baseline and analyses of subgroups or interaction effects reported		
Secondary	3. Sensitivity analyses are performed to check the robustness of results and the effects of key assumptions on definitions or outcomes		
*Results/reporting: 6 items (2 primary, 4 secondary)*			
Primary	1. Extensive presentation of results/authors describe the key components of their statistical approaches^a^		
Primary	2. Were confounder-adjusted estimates of treatment effects reported^b^		
Secondary	3. Flow chart explaining all exclusions and individuals screened or selected at each stage of defining the final sample		
Secondary	4. Was follow-up similar or accounted for between groups		
Secondary	5. Did the authors describe the statistical uncertainty of their findings		
Secondary	6. Was the extent of missing data reported		
*Discussion/interpretation: 4 items (2 primary, 2 Secondary)*			
Primary	1. Results consistent with known information or if not, was an explanation provided		
Primary	2. Are the observed treatment effects considered clinically meaningful		
Secondary	3. Discussion of possible biases and confounding factors, especially related to the observational nature of the study		
Secondary	4. Suggestions for future research to challenge, strengthen, or extend the study results		
*Conflict of interest: (1 primary item)*			
Primary	1. Potential conflicts of interest, including study funding, were stated		

^a^For a retrospective design, answering “no” to this item may suggest a “fatal flaw” using the methodology developed by ISPOR^14^

^b^For any study design, answering “no” to this item may suggest a “fatal flaw” using the methodology developed by ISPOR^14^

### Phase IV: Pilot application of the quality assessment tool

To pilot the tool and assess inter-rater variability, six papers from the Task Force’s literature review were randomly assigned for appraisal to two Task Force subgroups of nine members, Group A (n = 3 papers [15–17]) and Group B (n = 3 [18–20]). Three of the papers reviewed considered the relationship between adherence and asthma outcomes, and three the relationship between particle size or device type and outcomes. Participant Task Force members were not permitted to appraise papers that they had co-authored; papers were otherwise randomly allocated and reviews carried out independently.

Inter-rater variability also described as % rater agreement, was evaluated as the percentage concordance among raters at the individual sub-item, pooled primary sub-item, pooled secondary sub-item and combined primary/secondary sub-item level. For example, for a given sub-item, if four of the five raters deemed this sub-item as a “yes” whereas the remaining one rater deemed this sub-item as a “no,” then the inter-rater variability (% rater agreement) would be 4/5 = 80%. A percentage agreement that approaches 50% suggests large disagreement that is no better than chance whereas % agreement that approaches 100% suggests strong and full concordance across raters. Raters were invited to provide written feedback on usability of the checklist and to identify areas of difficulty in criterion interpretation or application. Potential areas of divergent interpretation were also explored through interactive discussion with two members of Group A to understand the rationale for their differences in opinions where they occurred.

The Task Force members next met at the American Thoracic Society Conference in May 2015 (Denver, Colorado, USA) to review the pilot results and enact necessary revisions. Inter-rater agreement was calculated and qualitative feedback invited from participating raters to identify opportunities to reduce inter-rater variability further. Redundancies in sub-items were identified through rater quality feedback and deleted.

Task Force rater feedback also recommended that secondary sub-items remained hidden until fulfillment of all the primary sub-items is confirmed in order to streamline the review process and avoid time being spent qualifying more subtle aspects of papers that may ultimately fail to meet the minimum requirement for guideline consideration.

A wider pilot of the refined checklist (Additional file 1: Table 2) was recommended to allow involvement of a larger number of raters (≥ 20) and to assess the impact of the implemented revisions and tool simplification on inter-rater agreement.

### Phase V: Extended pilot

Twenty-two participants were identified for the extended pilot via an open invitation emailed to all REG collaborators and members of EAACI (Additional file 1: Table 3). Six papers from the Task Force’s CER literature review were selected for appraisal [21–26] and assigned in pairs across three rater subgroups (Groups A [n = 7]; Group B [n = 8]; Group C [n = 7]). There were two papers on the relation between adherence and outcomes, two on the relation between persistence to therapy and healthcare resource utilization and two on the relation between particle size and outcomes.

Raters were asked to review the papers independently and to use an Excel-based version of the tool to record checklist fulfillment for each paper and then to return these to the Task Force coordinator via email for collation. A one-page user guide was developed to ensure all participating raters received (or had access to) standardized instructions as to the use of the tool.

As in the earlier pilot, raters were not allowed to review papers they had coauthored. Allocation of papers was otherwise random. As before, calculation of inter-rater variability (percentage agreement) was defined by consistent ratings (all raters scoring the same paper ‘Yes’ or all ‘No’) at a per-criterion level for each paper. Agreement results were then estimated for each paper at the item-, domain and global (all primary sub-items) level within each rater group and in sum across all rater groups.

The results of the pilot were reviewed by the Task Force members at a face-to-face meeting at the 2015 European Respiratory Society Conference (Amsterdam, The Netherlands). Further qualitative feedback was invited from pilot raters to identify remaining opportunities to remove redundancies, potential ambiguity and associated inter-rater variability.

The Task Force members implemented final rephrasing recommendations from the Phase V pilot and approved the tool for use for the primary Task Force CER literature review [8].

### Phase VI: development of an online tool

To aid ease of RELEVANT implementation for the wider Task Force literature review [8], an online version was developed using Google forms. The conversion of the tool to an online format minimized the potential for data mis-entry and incomplete data capture by use of drop down lists and questionnaire logic.

## Results

### Initial tool creation (Phase I–III)

Mapping the quality recommendations within the primary reference papers Berger et al. [14] and Roche et al. [13] resulted in an initial 43-item checklist (Additional file 2: Table 1). Implementation of the Task Force members’ recommendations for item-reduction narrowed the tool items to 25 quality sub-items distributed across seven core quality domains (Table 1).

### Tool appraisal and item reduction (Phases IV and V)

Evaluation of inter-rater agreement of the initial tool indicated wide variation (50–100%) in concordance across individual sub-items: < 60% for 8 of 25 sub-items; 60–67% for 10 of 25 sub-items and ≥ 80% for 7 of 25 sub-items. Domain-level agreement was similarly varied (50–100%). The qualitative feedback suggested this variation was largely driven by differences in semantic interpretation of some of the sub-items and could be addressed by further removal of any redundancies and careful rephrasing and further, e.g. eliminating subjective words (replacing “relevant” with “justified”) and splitting “double-headed” sub-items into separate, discrete sub-items.

The 22 raters involved in the extended pilot came from a wide range of countries (> 10) and included members of the REG collaborator group, EAACI Asthma Section Members, Global Initiative for Chronic Obstructive Lung Disease, Global Initiative for Asthma, the American Thoracic Society and the European Respiratory Society (Additional file 1: Table 3). Inter-rater agreement of the version of the tool used for the extended pilot found concordance in excess of 70% for 94% of primary sub-items and 93% of secondary sub-items (Table 2). When compared across the 3 groups (A–C), agreement on the primary quality sub-items varied from 64–100% to 55–100% for secondary sub-items.

**Table 2:** Extended Pilot Item level agreement summary

		Group A (%)	Group B (%)	Group C (%)
*a: Agreement across primary sub*-*items*				
1. Background	1.1. Clearly stated research question	79	100	86
2. Design	2.1. Population defined and justified	64	94	71
	2.2. Comparison groups defined and justified	93	71	79
	2.3. Setting defined and justified	93	100	93
3. Measures	3.1. (If relevant), exposure is clearly defined	93	71	76
	3.2. Primary outcomes clearly defined and measured	71	89	93
4. Analysis	4.1. Potential confounders are considered and adjusted for in the analysis, and reported	64	81	71
	4.2. Study groups are compared at baseline	79	79	79
5. Results	5.1. Results are clearly presented for all primary and secondary endpoints as well as confounders	79	94	71
6. Discussion/interpretation	6.1. Results consistent with known information or if not, an explanation is provided	86	100	86
	6.2. The clinical relevance of the results is discussed	85	88	93
7. Conflict of interests	7.1. Potential conflicts of interest, including study funding, are stated	79	100	93
*b: Agreement across secondary quality sub*-*items*				
1. Background	1.1. The research is based on a review of the background literature (ideal standard is a systematic review, but minimally citation of multiple [≥ 1] references in the introduction	88	100	100
2. Design	2.1. Clear written evidence of a priori protocol development and registration (e.g. in ENCePP or Clinicaltrials.gov online registries) and a priori statistical analysis plan	100	73	83
	2.2. The data source (or database), as described, contains adequate exposures (if relevant) and outcome variables to answer the research question	88	100	100
3. Measures	3.1. Sample size justifies the inclusion criteria and follow-up period for the primary outcome	88	71	88
4. Analysis	No secondary item	NA	NA	NA
5. Results	5.1. Flow chart explaining all exclusions and individuals screened or selected at each stage of defining the final sample	100	83	75
	5.2. Was follow-up similar or accounted for between groups (i.e. no unexplained differential loss to follow up)	100	55	58
	5.3. The authors describe the statistical uncertainty of their findings (e.g. p-values, confidence intervals)	100	100	83
	5.4. The extent of missing data is reported	75	88	100
6. Discussion/interpretation	6.1. Possible biases and/or confounding factors described	100	100	83
	6.2. Suggestions for future research provided (e.g. to challenge, strengthen, or extend the study results)	100	63	58
7. Conflict of interests	No secondary item	NA	NA	NA

Qualitative feedback from the pilot raters identified some remaining sub-items in the tool that could be further simplified to reduce potential inter-rater variability. Raters frequently commented on the ease of use of the tool, but noted the recurrent challenge of a lack of necessary information in the papers under appraisal. The Task Force members implemented the final rephrasing recommendations and finalized the checklist, with 21 items, as “RELEVANT” (Table 3).

**Table 3:** RELEVANT quality domains: primary sub-items (critical to satisfy minimum guideline requirements) and secondary sub-items (enabling further descriptive appraisal)

Quality domains and sub-items		Fulfilled (Y/N)
*Primary sub*-*items*		
1. Background	1.1. Clearly stated research question	
2. Design	2.1. Population defined	
	2.2. Comparison groups defined and justified	
3. Measures	3.1. (If relevant), exposure (e.g. treatment) is clearly defined	
	3.2. Primary outcomes defined	
4. Analysis	4.1. Potential confounders are addressed	
	4.2. Study groups are compared at baseline	
5. Results	5.1. Results are clearly presented for all primary and secondary endpoints as well as confounders	
6. Discussion/interpretation	6.1. Results consistent with known information or if not, an explanation is provided	
	6.2. The clinical relevance of the results is discussed	
7. Conflict of interests	7.1. Potential conflicts of interest, including study funding, are stated	
*Secondary sub*-*items*		
1. Background	1.1. The research is based on a review of the background literature (ideal standard is a systematic review)	
2. Design	2.1. Evidence of a priori design, e.g. protocol registration in a dedicated website	
	2.2. Population justified	
	2.3. The data source (or database), as described, contains adequate exposures (if relevant) and outcome variables to answer the research question	
	2.4. Setting justified	
3. Measures	3.1 Sample size/Power pre-specified	
4. Analysis	*No secondary item*	*NA*
5. Results	5.1. Flow chart explaining all exclusions and individuals screened or selected at each stage of defining the final sample	
	5.2. The authors describe the statistical uncertainty of their findings (e.g. *p* values, confidence intervals)	
	5.3. The extent of missing data is reported	
6. Discussion/interpretation	6.1. Possible biases and/or confounding factors described	
7. Conflict of interests	*No secondary item*	*NA*

### RELEVANT

The final tool—RELEVANT—guides systematic appraisal of the quality of published observational CER papers across seven domains: Background, Design, Measures, Analysis, Results, Discussion/Interpretation, Conflicts of Interest (Table 3). Raters must indicate fulfillment (Yes/No) of 11 quality sub-items across these seven domains, e.g. Background (Domain 1), Criterion 1.1: “Clearly stated research question” (Yes/No); Conflicts of Interest (Domain 7), Criterion 7.1: “Potential conflicts of interest, including study funding, are stated” (Yes/No). “RELEVANT quality” is defined as fulfillment of all 11 primary sub-items. Failure to meet any one primary criterion reflects a potential “fatal flaw” in a study’s design or, if failure reflects absence of the necessary detail, a lack of sufficient transparency in reporting. A fatal flaw is a concept defined by Berger et al. as an aspect of the: “design, execution, or analysis elements of the study that may significantly undermine the validity of the results [14]. If all primary sub-items are fulfilled, assessment of ten additional, secondary, parameters is prompted to enable further characterization of the relative strengths and weaknesses of the paper [8].

## Discussion

This is the first practical tool specifically developed to assist in the appraisal of published observational CER with the purpose of informing asthma guidelines and supporting decision-makers. Rater agreement was assessed in two pilots with the second, broader pilot returning robust results, reflecting the value of using Task Force expertise and early pilot rater feedback to revise the tool iteratively. We acknowledge that other tools such as the Grading of Recommendations, Assessment, Development and Evaluation (GRADE) exist for rating the quality of the best available evidence [2]. However, existing tools such as GRADE automatically downgrade observational study designs and upgrade randomized designs with the primary intent toward informing the quality of efficacy signals. The purpose of RELEVANT is more specific in that it focuses on observational CER studies that can potentially inform asthma guidelines and decision-makers. Further, RELEVANT attempts to weigh the benefits and harms of internal versus external validity, a domain lacking from GRADE and other tools that prioritize randomized designs and efficacy signals. Thus, within the specific case of real-world effectiveness study quality, broad tools that emphasize efficacy may not be fit for purpose.

RELEVANT enables quality appraisal of the *published literature* and so complements existing quality checklists, which have traditionally focused on quality markers for protocol and manuscript development and from a largely empirical (rather than practical/applied) perspective. Initial drafts of the tool used wording from the previous quality assessment literature, but the pilot work revealed the potential for misinterpretation unless very precise language was used and each criterion had a singular and specific focus. Refer to the Roche and colleagues companion manuscript for clinical applications and evaluations that use the RELEVANT tool [8].

RELEVANT was developed to assist clinical experts and guideline developers in appraising observational asthma CER study quality. Used in combination with other tools such as the GRADE, RELEVANT can help facilitate critical appraisal of observational studies as well as pragmatic trials and so contribute to a broader appraisal of the available asthma evidence base. It is a time efficient and user-friendly method of quality assessment and has potential utility as a teaching aid, manuscript development guide or as a tool for use by journal peer reviewers. Indeed, any researcher or clinician with the ability to critically read CER (e.g. basic knowledge of confounding in observational research) should find the tool self-explanatory and potentially useful as a publication checklist as well as a framework for literature appraisal.

RELEVANT development was an ancillary (although necessary) step towards completion of the original Task Force’s objective of conducting a quality appraisal of the observational asthma CER literature. As such, a quasi-pragmatic rather than entirely systematic and externally validated approach was taken. All primary and secondary sub-items featured in the tool were felt to be important (within the literature and by Task Force members), but expert opinion was used to differentiate between primary (mandatory) and secondary (complementary) sub-items to ensure RELEVANT was comprehensive but also relatively concise to aid in successful practical implementation. The final categorization was based on frequency of reference within the prior literature and on Task Force member consensus judgment. Prioritization and categorization of the primary/secondary sub-items within the checklist could be revisited and adapted for other purposes, as appropriate.

While RELEVANT is intended as a tool to assist in the quality appraisal of observational CER, RELEVANT assessments are influenced by the quality of reporting of the research as much as the by the inherent quality of the study itself. While this may result in an under-estimation of the quality of the published work (e.g. dismissal of a paper owing to a failure in reporting rather than in the research methodology) the Task Force members felt this was unavoidable. An alternative approach could involve contacting each author group and offering them the opportunity to provide more information where a quality limitation resulted from a lack of available data, but there was insufficient Task Force resources available to permit this approach. This emphasizes the need for accurate and comprehensive reporting and the interest of protocol registration.

While development of RELEVANT sought to remove subjective interpretation of the quality sub-items, the rater’s appraisal of the fulfillment of the quality markers is unavoidably open to rater opinion and judgment. In turn, the appropriateness of such judgment is inherently affected by raters’ experience and expertise with respect to observational CER. The majority of raters involved in the pilot work, however, were members of REG—a group that has a particular focus on real-life research methods—and so most participating raters would have had substantial prior knowledge and/or involvement in CER. This may have contributed to the high inter-rater agreement recorded especially during the extended pilot testing.

There is a need for moderation in the recommended potential uses of the checklist tool to avoid it being widely adopted for purposes other than those for which it was designed. However, there are also potential opportunities to use the tool in training exercises to educate fellows, journals editors and reviewers as to what constitutes quality in CER. If the tool were introduced within graduate-level training, it may also have the potential to help shape and inform the design of more appropriate observational CER in the future.

## Conclusions

RELEVANT is a user-friendly quality appraisal tool comprising 21 quality sub-items (11 primary; 10 secondary) across seven core quality domains. It was developed to support quality review of observational asthma CER for the purposes of the joint REG-EAACI Task Force literature review, but is also now used among Task Force members to support their peer review activities for respiratory journals and to guide the development of their own research papers. It is the first of its kind to support quality appraisal of published research and to have been developed through iterative feedback derived from pilot implementation and inter-rater agreement evaluation.

RELEVANT can be downloaded in Excel or pdf format from the REG [27] and EAACI [28] websites for use by guideline developers, researchers, medical writers, and other interested parties. Further, a RELEVANT tool user guide can be downloaded on the REG website [27].

## Additional files

**Additional file 1.** This file includes the goals of the Task Force, an intermediate item-reduce tool, and extended quality assessment tool pilot participants.

**Additional file 2.** This maps the quality recommendations within the primary reference papers Berger et al and Roche et al that resulted in an initial 43-item checklist.
