# Supplementary material for: The REal Life EVidence AssessmeNt Tool (RELEVANT): development of a novel quality assurance asset to rate observational comparative effectiveness research studies
Source: Clin Transl Allergy. 2019 Mar 27;9:21. doi: 10.1186/s13601-019-0256-9 (PMC6436213; doi:10.1186/s13601-019-0256-9)
Supplement: Supplementary file 1 — Additional file 1. This file includes the goals of the Task Force, an intermediate item-reduce tool, and extended quality assessment tool pilot participants. [file 13601_2019_256_MOESM1_ESM.docx]

**Supplementary Figure 1. Aims and objectives of the REG/EAACI asthma comparative effectiveness research quality appraisal Task Force**

In 2014, members of the international Respiratory Effectiveness Group (REG) and the European Academy of Allergy and Clinical Immunology (EAACI) formed a joint Task Force to respond to calls for observational comparative effectiveness research (CER) evidence to be better integrated into future guideline development and clinical decision-making. The Task Force was established to conduct a structured quality appraisal of the asthma comparative effectiveness literature with a view to synthesizing the available high-quality evidence for future guideline development and clinical decision-making. The Task Force members involved in the tool’s testing came from a wide range of countries (>10) and included members of the REG collaborator group, EAACI Asthma Section Members, the Global Initiative for Chronic Obstructive Lung Disease (GOLD), Global Initiative for Asthma (GINA) and Thoracic Societies (ATS and ERS)

#### Supplementary Table 2. Item reduced tool, following Task Force Feedback in May 2015 (at the ATS face-to-face meeting); used for wider pilot (7 Domains and 22 items)

| **BACKGROUND (1 Primary; 1 Supporting)** | |
| --- | --- |
| Primary | 1. Clearly stated research question |
| Supporting | 2. The research is based on a review of the background literature (ideal standard is a systematic review, but minimally citation of multiple [≥1] references in the introduction) |
| **DESIGN (3 primary; 2 supporting)** | |
| Primary | 1. Population defined and justified |
| Primary | 2. Comparison groups defined and justified |
| Primary | 3. Setting defined and justified |
| Supporting | 4. Clear written evidence of a priori protocol development and registration (e.g. in ENCePP or Clinicaltrials.gov online registries) and a priori statistical analysis plan |
| Supporting | 5. The data source (or database), as described, contains adequate exposures (if relevant) and outcome variables to answer the research question |
| **MEASURES (2 primary; 2 supporting)** | |
| Primary | 1. (If relevant), exposure is clearly defined |
| Primary | 2. Primary outcomes clearly defined and measured |
| Supporting | 3. Sample size justifies the inclusion criteria and follow-up period for the primary outcome |
| **ANALYSIS 2 items (2 primary; 0 supporting)** | |
| Primary | 1. Potential confounders are considered and adjusted for in the analysis, and reported |
| Primary | 2. Study groups are compared at baseline |
| **RESULTS 5 items (1 Primary; 4 Secondary)** | |
| Primary | 1. Results are clearly presented for all primary and secondary endpoints, and confounders |
| Supporting | 2. Flow chart explaining all exclusions and individuals screened or selected at each stage of defining the final sample |
| Supporting | 3. Was follow-up similar or accounted for between groups (i.e. no unexplained differential loss to follow up) |
| Supporting | 4. The authors describe the statistical uncertainty of their findings  (e.g. p-values, confidence intervals) |
| Supporting | 5. The extent of missing data is reported |
| **DISCUSSION / INTERPRETATION 4 items (2 Primary; 2 Supporting)** | |
| Primary | 1. Results consistent with known information or if not, was an explanation provided |
| Primary | 2 The clinical relevance of the results is discussed |
| Supporting | 3. Possible biases and/or confounding factors described |
| Supporting | 4. Suggestions for future research provided (e.g. to challenge, strengthen, or extend the study results) |
| **CONFLICT OF INTEREST 1 Item (1 Primary)** | |
| Primary | 1. Potential conflicts of interest, including study funding, were stated |

#### Supplementary Table 3. Extended Quality Assessment Tool Pilot Participants

| **MEMBER** | **COUNTRY** |
| --- | --- |
| **Eric Van Ganse*** | France |
| **Jennifer Quint*** | UK |
| **Jon Campbell*** | USA |
| **Nikolaos Papadopoulos*** | Greece, UK |
| **David Price*** | Singapore |
| **Arzu Bakirtas** | Turkey |
| **David Halpin** | UK |
| **Bernardino Alcazar Navarrette** | Spain |
| **Enrico Heffler** | Italy |
| **Helen Reddell** | Australia |
| **Joergen Vestbo** | Denmark, UK |
| **Juan José Soler Cataluna** | Spain |
| **Kostas Kostikas** | Switzerland |
| **Laurent Laforest** | France |
| **Ludger Klimek** | Germany |
| **Luis Caraballo** | Colombia |
| **Manon Belhassen** | France |
| **Matteo Bonini** | Italy |
| **Ömer Kalayci** | Turkey |
| **Piyameth Dilokthomsakul** | Thailand |
| **Sinthia Bosnic-Anticevich** | Australia |
| **Yee Vern Yong** | Malaysia |

*Task Force Member
